# Understanding the obesity dynamics by socioeconomic status in Colombian and Mexican cities using a system dynamics model

**DOI:** 10.1016/j.heliyon.2024.e39921

**Published:** 2024-10-29

**Authors:** Jose D. Meisel, Valentina Esguerra, Carolina Pérez Ferrer, Ivana Stankov, Felipe Montes, Natalia Tumas, Usama Bilal, Juan A. Valdivia, Ana V. Diez Roux, Olga L. Sarmiento

**Affiliations:** a Facultad de Ingeniería, Universidad de Ibagué, Carrera 22 Calle 67, 730001, Ibagué, Colombia; b Social and Health Complexity Center, Bogotá, Colombia; c CONACyT-Instituto Nacional de Salud Pública, Cerrada de Fray Pedro de Gante 50, 14080, Mexico City, Mexico; d Urban Health Collaborative, Dornsife School of Public Health, Drexel University, 3600 Market St, 7th floor, Philadelphia, PA, 19104, USA; e UniSA Allied Health and Human Performance, University of South Australia, Adelaide, South Australia, Australia; f Department of Industrial Engineering, Social and Health Complexity Center, Universidad de los Andes, Carrera 1 Este No. 19A-40, Bogotá, Colombia; g Centro de Investigaciones y Estudios sobre Cultura y Sociedad(CIECS), Consejo Nacional de Investigaciones Científicas y Técnicas(CONICET) y Universidad Nacional de Córdoba (UNC), Córdoba, Argentina; h Johns Hopkins University - Universitat Pompeu Fabra Public PolicyCenter (JHU-UPF PPC), UPF- Barcelona School of Management (UPF-BSM),Barcelona, Spain; i Department of Epidemiology and Biostatistics, Dornsife School of Public Health, Drexel University, 3215 Market St, 5th floor, Philadelphia, PA, 19104, USA; j Departamento de Física, Facultad de Ciencias, Universidad de Chile, Las Palmeras, 3425, Ñuñoa Santiago, Chile; k Centro para el Desarrollo de la Nanociencia y la Nanotecnología, CEDENNA, Santiago, Chile; l Department of Public Health, School of Medicine, Universidad de los Andes, Carrera 1 Este No. 19A-40, Bogotá, Colombia

**Keywords:** Complex systems, Epidemiology, Obesity transition, Public health, System dynamics model, System science

## Abstract

**Purpose:**

This paper aims to enrich understanding of the obesity transition among socioeconomic status (SES) strata by gender and age in cities of Colombia and Mexico. The study uses harmonized data from the Salud Urbana en América Latina (SALURBAL) study.

**Methods:**

A population-level system dynamics model was developed using 2010 and 2015 data from Colombia and 2012 and 2016 data from Mexico (national health surveys). The model simulates the prevalence of different BMI categories (i.e., not overweight, overweight, obese) stratified by gender, age, and SES, in the SALURBAL cities (aggregated to the country level) of Colombia and Mexico from 2010 to 2050. Sample sizes for Colombia in 2010 and Mexico in 2012 were 7420 and 5785 children (<5 years), 21601 and 14413 children and adolescents (5–17 years), and 46597 and 20464 adults (18–64 years), respectively. Sample sizes for Colombia in 2015 and Mexico in 2016 were 4450 and 907 children, 12468 and 2350 children and adolescents, and 90430 and 3413 adults, respectively.

**Results:**

For men in Colombia and Mexico, the burden of obesity is projected to increase among lower SES adults over time. Colombian women show similar patterns observed in men but the burden of obesity was already greater in the lower SES groups as early as 2012. In Mexican women, the burden of obesity in 2012 is higher in the lower SES population; however, the prevalence of obesity is projected to increase at a faster rate in the higher SES population. Patterns for children aged 0–14 years differed by gender and country.

**Conclusions:**

The model suggests that the prevalence of obesity among SES strata by age and gender in SALURBAL cities of Colombia and Mexico are likely to change over time, and predicts their possible evolution through the different stages of the obesity transition.

## Highlights

• Projected obesity transition patterns differed in Colombia and Mexico.
• Overweight and obesity were projected to increase more in Colombia than in Mexico.
• For men in Colombia and Mexico, the burden of obesity tends to shift towards those with lower socioeconomic status.
• For women, different trends in the prevalence of obesity were observed in Colombia and Mexico.
• Efforts to prevent obesity in Colombia and Mexico should consider equity criteria.

## 1 Introduction

Rates of obesity worldwide have reached epidemic proportions and constitute a leading public health challenge in several countries [1]. Since 1975, the global prevalence of overweight and obesity has increased [2], and recent estimates suggest that over a third of the world's population have overweight or obesity [3,4]. The age-standardized prevalence of obesity increased from 4.6 % in 1980 to 14.0 % in 2019 [5]. Epidemiological studies have shown that elevated body mass index (BMI) is associated with several chronic diseases, including non-alcoholic fatty liver disease (NAFLD), cardiovascular disease, diabetes, various cancers, musculoskeletal disorders, chronic kidney disease, and mental health, thereby diminishing quality of life and contributing to increased medical costs [5]. Furthermore, the stigma around obesity hinders educational and work opportunities for people living with this condition [6]. In 2015, overweight and obesity represented the lead risk factor for 7.1 % of all deaths recorded that year, while in 2019, high BMI was the fifth leading risk factor for attributable deaths (with approximately 5 million deaths worldwide) [7].

Prior evidence in low- and middle-income countries (LMIC) has shown that obesity is increasingly affecting lower socioeconomic status (SES) groups [[8], [9], [10]]. For example, obesity prevalence tends to be higher among more advantaged women in low-income countries but as countries develop, the burden of obesity changes towards more socially disadvantaged women. In the case of men, these transitions also occur but at later stages [2,8,[10], [11], [12]]. Similar patterns have been observed when stratifying countries using the human development index (HDI) and different SES indicators (e.g., education, employment, material belonging, area level indicators) [13]. These changes in the distribution of obesity by gender, age, and SES over time have recently been referred to by Jaacks et al. [14] as the obesity transition. These shifts, especially in LMIC countries, pose great concerns for health systems and the economy [15,16].

Obesity has received increasing attention from governments around the globe, including Latin American countries. Over the course of the last decade, countries in Latin America have implemented a range of national policies and health promotion programs to improve nutrition and physical activity patterns [[17], [18], [19], [20]]. Despite this, overweight and obesity are projected to affect 50 % of men and 60 % of women in Latin America by 2030 [2]. Concurrently, several countries in Latin America and the Caribbean are showing inverse or curvilinear associations between obesity and SES (e.g. Chile [21], Brazil [22,23], Curacao [24], Colombia [[25], [26], [27]], Mexico [28], and Argentina [29]).

Given the dynamic and complex nature of obesity, calls have been made for the use of complex systems methods, such as system dynamics (SD) models, to better understand and address the public health challenges posed by the obesity epidemic [[30], [31], [32]]. Complex systems approaches have several notable advantages over statistical models, as they can account for nonlinearities, focus on the dynamic interaction between actors, and take into account feedback loops to study emergent phenomena over time, including the unintended impacts of interventions [[33], [34], [35], [36], [37], [38], [39], [40], [41]]. Various studies have been conducted to explore the relationship between obesity and a range of political, economic, environmental, social, cultural, digital, behavioral, and biological factors [31,42]. To date, however, most studies that have focused on obesity transitions and obesity patterns by SES have applied statistical models instead of complex systems approaches [31,42].

System dynamics modeling and other complex systems methods have been utilized by several studies to understand obesity and its associated factors [31,[43], [44], [45], [46]]. However, research specifically investigating the obesity transition and its dynamics from a complex systems perspective is relatively scarce [[25], [26], [27],^47^]. Examples of such research include, Fallah-Fini et al. [48] and Fallah-Fini et al. [49], employing population SD models to quantify the energy imbalance gap (EIG) by gender and racial subpopulations among adults in the US and New Zealand, respectively. Sabounchi et al. [50], developed a population SD model focusing on obesity among women as a decision tool for policymakers. Zainal Abidin et al. [51] applied an SD optimization model to simulate the effect of changes in the eating behavior of British children (aged 2–15 years). Additionally, other studies have employed SD models to examine the mechanisms driving childhood overweight and obesity and/or testing of preventive interventions [52,53]. In the Caribbean region, Guariguata et al. [54] proposed a SD model to evaluate the changes in physical activity and dietary habits necessary to meet the global targets set by the WHO Global Action Plan. In the Latin American region, some studies have been developed to study the obesity transition by age, gender, and SES at country, regional, and department levels in Colombia [[25], [26], [27]]. Moreover, of these studies, none have applied complex system approaches to model the obesity transition across multiple countries.

This study aims to enrich understandings of the dynamics of obesity by developing a model that allows the exploration of dynamics driving the prevalence of and temporal changes in obesity by gender, age, and SES in the cities of Latin America. Data for cities in Colombia and Mexico was derived from the Urban Health in Latin America project (‘Salud Urbana en América Latina’; SALURBAL), which is an international collaborative project that studies how urban environments and urban policies impact the health of city residents and environmental sustainability throughout Latin America. SALURBAL aims to inform policies and interventions to create healthier, more equitable, and more sustainable cities worldwide [55].

## 2 Methods

To understand the changes in the obesity dynamics in Colombian and Mexican cities over time, a calibrated and validated population-level SD model [25,26,56] was used to study the prevalence of BMI categories across SES strata by gender and age. The model projected obesity prevalence ratios comparing the lowest to the highest SES groups through the year 2050.

### 2.1 Data

This study uses the most recent available data harmonized by the SALURBAL Project [57]. Data were derived from the 2010 and 2015 ‘Encuesta Nacional de la Situación Nutricional en Colombia’ (ENSIN) Survey for Colombia, and from the 2012 and 2016 ‘Encuesta Nacional de Salud y Nutrición’ (ENSANUT) Survey for Mexico. Specifically, data from SALURBAL-defined cities in Colombia and Mexico with at least two repeat measures of BMI over time was used. Cities in SALURBAL were defined as clusters of administratively-defined areas (i.e., municipios). SALURBAL has compiled and harmonized data on cities with a population above 100 000. The sample comprised 7420 and 5785 children younger than 5 years, 21601 and 14413 children and adolescents aged 5–17 years, and 46597 and 20464 adults aged 18–63 years from Colombia in 2010 and Mexico in 2012, respectively. The corresponding samples for Colombia in 2015 and Mexico in 2016, respectively, were 4450 and 907 (<5 years), 12468 and 2350 (5–17 years), and 90430 and 3413 (18–64 years). To understand the dynamics of obesity in Colombia and Mexico, the SD model for SALURBAL cities was run by aggregating the data for all SALURBAL cities in each country. The study also used data on population size, mortality, fertility, and socioeconomic status. The main data and variables used to inform the models are described in Table 1.

**Table 1:** Variables and data sources used in the system dynamics model.

Variable	Source
***Prevalence rates by BMI category, age, gender, and SES***	
BMI for age and sex z-score for children and adolescents aged 0 to 17 by mother's education level (as a proxy of SES): Not overweight: BMI for age and sex z-score ≤1 standard deviation. Overweight: BMI for age and sex z-score >1 standard deviation and ≤2 standard deviations. Obesity: BMI for age and sex z-scores >2 standard deviations.	For Colombia, ENSIN 2010 and 2015 For Mexico, ENSANUT 2012 and 2016 WHO child growth standards [58] and references [59]
BMI category cut-points by age, gender, and education level (as a proxy of SES) for adults aged 18 to 64 years: Not overweight: BMI <25 kg/m^2^ Overweight: BMI ≥25 and < 30 kg/m^2^ Obesity: BMI ≥30 kg/m^2^	For Colombia, ENSIN 2010 and 2015 For Mexico, ENSANUT 2012 and 2016 WHO cut-off points [60]
***Population composition***	
Population size by age, gender, and SES	For Colombia, Colombian National Department of Statistics (DANE). Estimations and population projections for 2005–2017 [61], and for México, Instituto Nacional de Estadística, Geografía e Informática (INEGI) and the Consejo Nacional de Población (CONAPO) [[62], [63], [64]]
Mortality rates (deaths per population per year, for all-cause mortality) by age group and gender	For Colombia, DANE. Data for the years 1979–2015 [65], and for México, INEGI. Data for the years 1990–2015 [66]
Fertility rate (births for each woman per year)	For Colombia, World Data Bank. World Development Indicators (1960–2018) [67], and for México, INEGI [68]. Projections for 2018–2050. Forecast series using the HoltWinters no seasonal method in EViews 10 (Quantitative Micro Software, LLC)
***SES indicator***	
For children and adolescents aged 0 to 17 years: Educational attainment of the mother: Less than primary completed, primary completed, secondary completed, or university completed	For Colombia, ENSIN 2010 and 2015 For Mexico, ENSANUT 2012 and 2016
For adults aged 18 to 64 years: Educational attainment of the individual: Less than primary completed, primary completed, secondary completed, or university completed	For Colombia, ENSIN 2010 and 2015 For Mexico, ENSANUT 2012 and 2016

### 2.2 Model of obesity dynamics

**Fig. 1:**
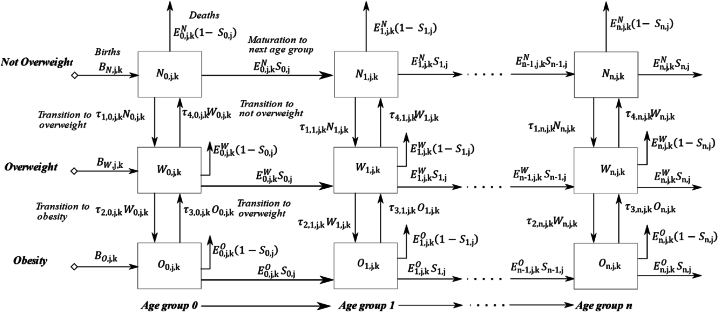
Overview of the system dynamics model structure.

The model includes aging chains for three BMI categories (*not overweight*, *overweight*, and *obesity*) based on the WHO standards and references (Table 1) [59,60]. Aging chains are used to allow disaggregation of the total stock (population aged 0–59) into multiple categories (age groups by BMI category and SES group) and to track the age structure of the stock. The Colombian population was divided into 5-year age groups because available data have an interval of 5 years (2010 and 2015). A validated heuristic [25,26,56] was used to estimate the transference rates (TRs) aggregated over 5 years, based on two time points. Similarly, the Mexican population was divided into 4-year age groups because available data have an interval of 4 years (2012 and 2016), with TRs aggregated over this 4year interval. These TRs are included in the model to simulate the dynamics of obesity by age, gender, and SES over time. The population aging structure also includes births and deaths for each cohort, with death rates differing according to age group and gender. The same mortality rate was assumed for each BMI category, in each age group, because mortality rate data further stratified by gender and SES was unavailable. Fig. 1 summarizes the SD model structure.

In Fig. 1, *i* ∈ (0, ….,n) represents the age groups (for Colombia, the model used 12 age groups in intervals of 5 years, and for Mexico, the model used 15 age groups in intervals of 4 years), *j ∈ (1=men, 2=women)* represents the gender*,* and *k ∈ (1= less than primary completed or primary completed, 2= secondary completed, and 3= university completed)* represents educational attainment, which is used as a proxy for the SES group of the simulated population.

*N*_*i,j,k*_*(t)*, *W*_*i,j,k*_*(t)*, and *O*_*i,j,k*_*(t)* are the populations of *not-overweight*, *overweight*, and *obesity* individuals in age group *i*, gender *j,* and SES *k* respectively, at time *t* (unit: people); *B*_*N,j,k*_*(t), B*_*W,j,k*_*(t),* and *B*_*O,j,k*_*(t)* are the births for each BMI category, gender *j,* and SES *k* of the first age group at time *t* (unit: people per year); and *E*_*i,j,k*_^*N*^*(t), E*_*i,j,k*_^*W*^*(t),* and *E*_*i,j,k*_^*O*^*(t)* are the exit rates of individuals per year for each age group *i,* gender *j*, SES *k*, and BMI category at time t (unit: people per year). The exit rate corresponds to the total number of individuals per year that leave each age group, by BMI category. Two types of individuals can leave each age group: *E*_*i,j,k*_^*N*^*(t)S*_*i,j*_*, E*_*i,j,k*_^*W*^*(t)S*_*i,j*_*,* and *E*_*i,j,k*_^*O*^*(t)S*_*i,j*_*,* individuals who mature into the next age group for each BMI category, age group *i*, gender *j*, and SES group k, respectively, at time *t* (unit: people per year), and *E*_*i,j,k*_^*N*^*(t)(1-S*_*i,j*_*), E*_*i,j,k*_^*W*^*(t)(1-S*_*i,j*_*),* and *E*_*i,j,k*_^*O*^*(t)(1-S*_*i,j*_*),* individuals who die in each BMI category, age group *I,* gender *j*, and SES group *k*, respectively, at time *t* (unit: people per year); *S*_*i,j*_ is the survival fraction per year for each age group *i, and* gender *j* (unit: % per year) (Fig. 1).

Finally, *τ*_*1,i,j,k*_ and *τ*_*2,i,j,k*_ are the TRs that correspond to the fraction of individuals per year from the *not overweight* and the *overweight* categories that become *overweight* and *obesity* for each age group *i,* gender *j*, and SES *k*, respectively (unit: % per year). *τ*_*3,i,j,k*_ and *τ*_*4,i,j,k*_ are the TRs of individuals from the *obesity* to the *overweight* category and from the *overweight* to the *not overweight* category, respectively, from each age group *i,* gender *j*, and SES *k* (unit: % per year) (Fig. 1).

### 2.3 Estimation of transference rates between BMI categories

A validated heuristic [25,26,56] was used to approximate the prevalence rates by BMI category in 2015 for Colombia and 2016 for Mexico, for each age group, gender, and SES group independently using the system of equations (1) described in Supplementary File. Then, the heuristic estimates the TRs by BMI category, age, gender, and SES using a minimization process. This process involves minimizing the quadratic difference between prevalence rates by BMI category, age, gender, and SES informed by health surveys (ENSIN and ENSANUT) in 2015 for Colombia and 2016 for Mexico and the prevalence rates estimated by the SD model in the same years for each country. For the purpose of the study, the TRs were assumed uniform within each age group but differ across age groups. These estimated TRs are included in the SD model, as shown in Fig. 1, to simulate the prevalence rates by BMI category, age, gender, and SES over time. These TRs are also assumed to be stable over the simulated timeframe given the lack of additional longitudinal data needed to calculate the TRs by BMI category, age, gender, and SES from one year to the next (only harmonized data for ENSIN 2010 and 2015 and for ENSANUT 2012 and 2016 were available).

### 2.4 Model of obesity dynamics by BMI and SES

To examine the obesity transition by SES over time, the level of educational attainment variable was used as a proxy for SES. For individuals aged 0 to 17, the mother's level of education and for adults the individual's educational attainment was used. The model in this study simulated population-level trends in the prevalence of each BMI category by age, gender, and SES until 2050. These projections were also used to compare the lowest SES to the highest SES groups using the obesity prevalence ratio (PR). The PR measures the ratio of probability, for each gender *j*, of obesity in the lowest SES group to the probability of obesity in the highest SES group for individuals aged 20–59 years, as expressed by:

PRj(t)=∑i=411Oi,j,1(t)∑i=411(Ni,j,1(t)+Wi,j,1(t)+Oi,j,1(t))∑i=411Oi,j,3(t)∑i=411(Ni,j,3(t)+Wi,j,3(t)+Oi,j,3(t))

Where, *N*_*i,j,1*_*, W*_*i,j,1*_*, O*_*i,j,1*_ represent all individuals with *not overweight*, *overweight*, and *obesity* with lower SES, respectively, in age group *i* and gender *j*, and *N*_*i,j,3*_*, W*_*i,j,3*_*, O*_*i,j,3*_ represent all individuals with *not overweight*, *overweight*, and *obesity* of higher SES, respectively, in age group *i* and gender *j*. A PR value greater than one indicate that the burden of obesity is greater among adults with lower SES. This study assumed individuals do not transition between SES groups given the lack of available data to estimate these TRs by BMI category, age, and gender. The data processing was conducted using Mathematica 11 (Wolfram Research, Inc) and all simulations were run on iThink 9.0.2 (ISEE Systems, Inc). A detailed description of the SD model and the heuristic is available in prior publications [25,26,56]. Key assumptions of the model are summarized in Table S3 of the Supplementary File.

### 2.5 Validation of the model

To assess the suitability of the SD model in achieving its intended purpose [69] a behavior reproduction method was used. The two-sample Kolmogorov–Smirnov test was used to compare the prevalence rates by BMI category, age, gender, and SES reported by the health surveys in 2015 for Colombia and 2016 for Mexico and the estimated prevalence rates estimated by the SD model in the same years for each country. The results and a more detailed explanation of the test are available in Supplementary File, Section 3.

## 3 Results

**Fig. 2:**
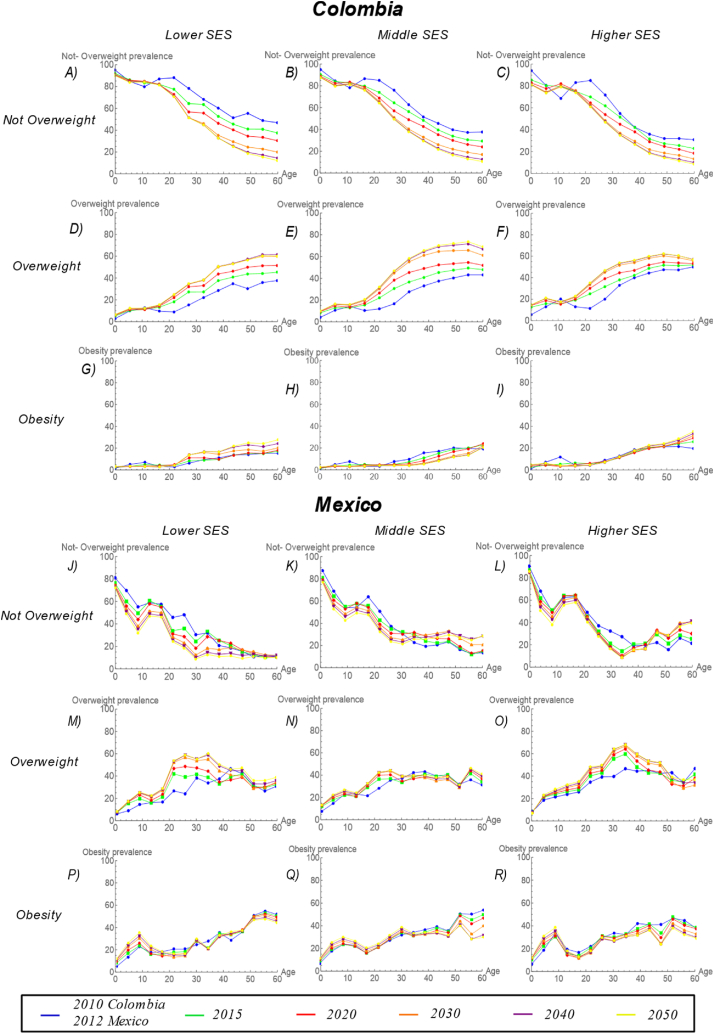
**Prevalence rates projected to 2050 for men by BMI category, age, and SES in Colombian and Mexican cities.** For Colombian men: (A) Not overweight, lowest SES; (B) not overweight, middle SES; (C) not overweight, highest SES; (D) overweight, lowest SES; (E) overweight, middle SES; (F) overweight, highest SES; (G) obesity, lowest SES; (H) obesity, middle SES; and (I) obesity, highest SES. For Mexican men: (J) not overweight, lowest SES; (K) not overweight, middle SES; (L) not overweight, highest SES; (M) overweight, lowest SES; (N) overweight, middle SES; (O) overweight, highest SES; (P) obesity, lowest SES; (Q) obesity, middle SES; and (R) obesity, highest SES. Blue = 2010 (for Colombia) and 2012 (for Mexico); Green = 2015; Red = 2020; Orange = 2030; Purple = 2040; Yellow = 2050. (For interpretation of the references to colour in this figure legend, the reader is referred to the Web version of this article.)

Among men, projected obesity transition patterns differed in Colombia and Mexico (Fig. 2). In general, overweight and obesity were projected to increase more over time in Colombia than in Mexico. In Colombian men, between 2010 and 2050, projections suggest no major changes over time in obesity or overweight prevalence among those aged 0–14 years in any SES group. However, in the 20–59 age group, not overweight prevalence decreased with approximately similar patterns by SES (Fig. 2A, B, and 2C), and overweight and obesity prevalence increased over time but the increases were smaller in the highest SES group compared to the other two SES groups (Fig. 2D, E, 2F, 2G, 2H, and 2I). Specifically, the prevalence of overweight in men aged 20–59 years in the lowest, middle, and highest SES groups increased from 34.8 % to 54.9 %, from 39.8 % to 61.5 %, and from 43.8 % to 55.2 %, respectively (Fig. 2D, E, and 2F). The prevalence of obesity in men aged 20–59 years increased from 13.4 % to 26.2 %, from 18.3 % to 22.1 %, and from 21.9 % to 30.5 %, in the lower, middle, and higher SES groups respectively (Fig. 2G, H, and 2I).

In Mexican men, between 2012 and 2050, the prevalence of not overweight is projected to decrease (Fig. 2J, K, and 2L), and the prevalence of overweight and obesity is projected to increase over time with approximately similar patterns by SES, among those aged 0–14 (Fig. 2M, N, 2O, 2P, 2Q, and 2R). Overweight prevalence increased from 14.7 % to 22.8 %, from 15.1 % to 22 %, and from 16.6 % to 20.7 %, and obesity prevalence increased from 11.8 % to 24 %, from 12.5 % to 20.3 %, and from 18 % to 27 % in lower, middle and higher SES groups, respectively. In the 20–59 age group, the prevalence in overweight increased over time with larger increases in the lower SES groups but the prevalence of obesity did not change much (and in fact declined somewhat) with no strong SES patterning. Specifically, the prevalence of overweight in the lowest, middle, and highest SES groups increased from 37.7 % to 53.2 %, from 38.8 % to 42.5 %, and from 40.3 % to 51 %, in the lower, middle, and higher SES groups respectively (Fig. 2M, N, and 2O). The prevalence of obesity changed from 35.5 % to 32.6 %, from 35.3 % to 32 %, and from 35.4 % to 28.6 %, in the lower middle and higher SES groups, respectively (Fig. 2P, Q, and 2R).

**Fig. 3:**
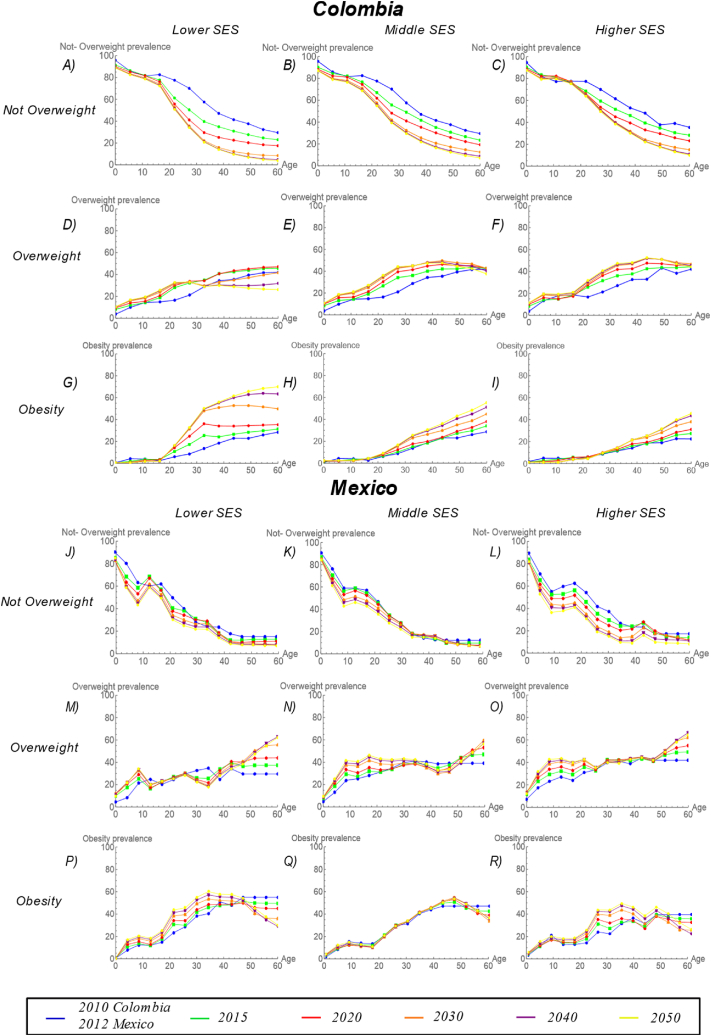
**Prevalence rates projected to 2050 for women by BMI category, age, and SES in Colombian and Mexican cities.** For Colombian women: (A) Not overweight, lowest SES; (B) not overweight, middle SES; (C) not overweight, highest SES; (D) overweight, lowest SES; (E) overweight, middle SES; (F) overweight, highest SES; (G) obesity, lowest SES; (H) obesity, middle SES; and (I) obesity, highest SES. For Mexican women: (J) not overweight, lowest SES; (K) not overweight, middle SES; (L) not overweight, highest SES; (M) overweight, lowest SES; (N) overweight, middle SES; (O) overweight, highest SES; (P) obesity, lowest SES; (Q) obesity, middle SES; and (R) obesity, highest SES. Blue = 2010 (for Colombia) and 2012 (for Mexico); Green = 2015; Red = 2020; Orange = 2030; Purple = 2040; Yellow = 2050. (For interpretation of the references to colour in this figure legend, the reader is referred to the Web version of this article.)

As in the case of men, overweight and obesity rates in women were projected to increase more in Colombia than in Mexico. Among women, in Colombia, between 2010 and 2050, there were no large changes over time in the prevalence of obesity among children and adolescents aged 0–14 years (Fig. 3G, H, and 3I). However, in this age group, the prevalence of not overweight is projected to decrease (Fig. 3A, B, and 3C) and the prevalence of overweight is projected to increase over time with approximately similar patterns by SES (Fig. 3D, E, and 3F). Specifically, the prevalence of overweight increased from 10.2 % to 23.5 %, from 10.6 % to 20 %, and from 13 to 17.1 % in lower, middle and higher SES groups respectively. Among women aged 20–59 years, overweight and obesity prevalence increased over time. Increases in overweight prevalence were larger in the higher SES groups but increases in obesity prevalence were higher in the lower SES group (Fig. 3G, H, and 3I). Specifically, the prevalence of overweight among women in the lowest, middle, and highest SES groups is projected to change from 36.2 % to 32.7 %, from 39.1 % to 42.2 %, and from 38 % to 46.6 %, respectively (Fig. 3D, E, and 3F). In the case of obesity prevalence, the differences in changes over time by SES groups were even more prominent than in men: the prevalence of obesity increased from 29.3 % to 58.7 %, from 28.5 % to 45 %, and from 24 % to 36.6 % in the lower, middle and higher SES groups, respectively (Fig. 3G, H, and 3I).

Among women in Mexico between 2012 and 2050, the prevalence of not overweight is projected to decrease at a greater rate in the highest compared to the others SES (Fig. 3J, K, and 3L). Furthermore, there were no large changes in the prevalence of obesity in the 0-14 year-old group (except for slightly larger increases in the lowest SES group which increased from 8.5 % to 15.1 %). Overweight rates increased in children aged 0–14 with no clear pattern by SES. Increases were from 15.8 % to 27.1 %, from 14.2 % to 25.5 %, and from 15 % to 29.1 % in lower, middle, and higher SES groups, respectively (Fig. 3M, N, and 3O). In the 20–59 age group, the prevalence of overweight increased similarly in the lowest, middle, and highest SES groups (from 33.0 % to 38.3 %, from 36.1 % to 39.5 % and from 38.0 % to 43.6 %, respectively) (Fig. 3M, N, and 3O). The prevalence of obesity is projected to increase at a greater rate in the highest compared to the others SES (the prevalence of obesity among women aged 20–59 years increased from 44.5 % to 48.6 %, from 39.6 % to 40.9 %, and from 31.6 % to 41.1 % in lower, middle, and higher SES groups, respectively) (Fig. 3P, Q, and 3R).

**Fig. 4:**
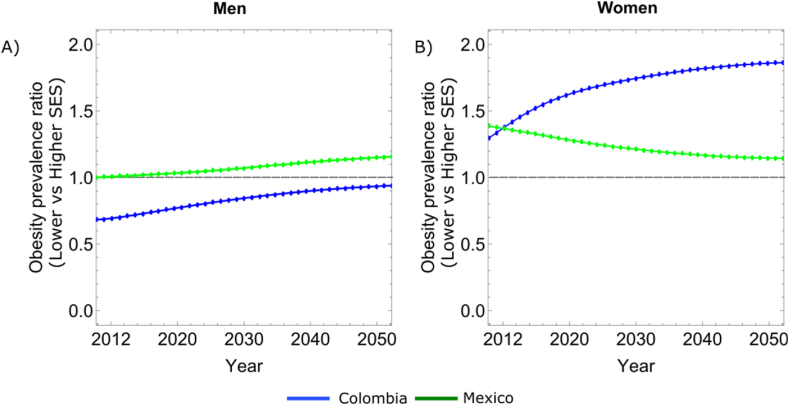
**Obesity prevalence ratio (PR) among adults aged 20**–**59 years by gender.** (A) Colombian and Mexico Men. (B) Colombia and Mexico Women. PRs above 1 represent a higher prevalence in lower SES groups, and PRs below one represent a higher prevalence in higher SES groups.

The simulated obesity PRs comparing lower to higher SES for men and women indicate that Mexico and Colombia are at different stages of the obesity transition. For both Colombia and Mexico, the burden of obesity among men tends to shift over time towards those with lower SES as indicated by the positive slopes in Fig. 4A. The projections indicate, for both countries, that obesity among lower SES men is increasing at a faster rate than obesity among higher SES men. However, the prevalence of obesity in Colombia is projected to remain highest among men with higher SES, at least up to 2050, while for Mexico the PR surpassed 1 in 2018 (Fig. 4A). For women, different trends were observed. Specifically, among Colombian women, the burden of obesity is projected to continue shifting towards those with lower SES. In Mexico, the 2012 obesity PR among women suggests that the burden of obesity was greatest in the lowest SES group but this ratio declines over time given that the prevalence of obesity was projected to increase at a slower rate in this SES group than in the higher SES group (Fig. 4B).

## 4 Discussion

Using the conceptual model proposed by Jaacks et al. [14] and empirical data obtained from the SALURBAL project, this paper analyzed the dynamics of obesity and found variations in the projected burden of obesity by gender, age, and SES in Colombia and Mexico. Overall, overweight and obesity were projected to increase more in Colombia than in Mexico. For men in Colombia and Mexico, the projections suggest that the burden of obesity will likely increase in the lower SES group over time, such that by the year 2050 the PR of obesity in lower vs higher SES men will exceed 1 in Mexico and approach 1 in Colombia (Fig. 4A). However, for women, patterns of obesity differ across the two countries. While Colombian women are projected to have similar patterns in the obesity PR as Colombian men (with the PR for low vs higher SES women increasing over time), the findings suggest that they have already experienced an obesity transition by SES (i.e. obesity rates were higher in the lower SES groups as early as 2012), whereas for Colombian men the PR is below 1 across the study period and only approaches 1 in 2050 (Fig. 4A and 4B). In the case of Mexican women, although the burden of obesity in 2012 is higher in the lower SES group, the projections indicate that the prevalence of obesity will increase faster in the higher SES population, resulting in a decline in the PR over time. This is in contrast to an increase in the PR among Mexican men (Fig. 4A and 4B). Notably, increases in the prevalence of overweight girls aged 0–14 years were projected in both countries with no SES patterning and an increase in obesity was observed in low SES Mexican girls. In boys, no projected changes in overweight and obesity rates were observed in Colombia but both obesity and overweight prevalence were projected to increase in Mexican boys (Fig. 2, Fig. 3).

The findings are consistent with existing research [9,14,26,70]. Specifically, the results align with the conceptual model of obesity transition proposed by Jaacks et al. [14]. Jaacks et al. [14] show that in 2016, Colombia and Mexico were in stage 2 of the obesity transition, a stage characterized by high obesity prevalence among adults (between 20 and 40 %), a smaller increase in the obesity prevalence among children, and a reduction in the differences in obesity prevalence by gender and SES (especially among women), compared to results observed in stage 1. In the case of Colombia, Jaacks et al. [14] show that, from 2005 to 2010, the highest increases in obesity prevalence were among the lowest SES group compared with the highest SES group. The simulations suggest that Colombia is moving towards stage 3 of the obesity transition, which is characterized by a higher obesity prevalence among those with lower SES than those with higher SES (Fig. 4A and 4B). However, projections regarding increases in SES differences over time in Colombian women do not align with Jaacks et al. [14], but are in line with prior studies in this country [25,26]. Meisel et al. (2020) show that Colombia is experiencing an obesity transition where the burden of obesity is shifting to lower SES adults, especially among women. In the case of Mexico, Jaacks et al. [14] reported that Mexico was at stage 2 of the obesity transition in 2016. However, the results of this study suggest that Mexico is between stages 2 and 3. The simulation results indicate that the burden of obesity, for men, will likely increase in the lower SES group over time, but for women, although the burden of obesity in 2012 is higher in the lower SES group, the prevalence of obesity will increase faster in the higher SES population over time (Fig. 4A and 4B). These results for Mexico also align with Perez-Ferrer et al. [28] who show that obesity prevalence in Mexico continued to increase among all SES groups from 2012 to 2016 but the highest burden of obesity was in women of the lowest SES.

This study provides a general overview of how Mexico and Colombia have and will likely evolve through the different stages of the obesity transition until 2050. Specifically, the results highlight possible anticipated patterns in the obesity transition by age, gender, and SES that are crucial to monitoring the implementation of policies within these countries. To place this result in the context of specific policies, it is important to recognize that Mexico and Colombia have taken multiple actions to prevent obesity. In Mexico, these have included the sugar-sweetened beverage tax, the front-of-pack warning labels, and school food guidelines [[71], [72], [73], [74], [75]]. In Colombia, key policies have included the national policy on food and nutrition security and the obesity law (National law 1355/2009), which seeks to promote healthy habits and lifestyles. Recently, Colombia implemented policies related to the sugar-sweetened beverage tax and front -of-pack warning labels. Taxation of unhealthy foods and beverages has been shown to reduce obesity across socioeconomic categories [[76], [77], [78]]. However, it is important for policymakers to consider how the effectiveness of obesity prevention interventions may differ by demographic characteristics and across SES [79]. For example, labeling policies may be most effective among individuals with higher educational attainment, thereby increasing inequities in ultra-processed foods (UPF) purchasing in settings where UPF purchasing is highest among low-income households, such as Chile [76]. The model highlights the need for governments to continue developing and investing in comprehensive public health policy packages directed toward addressing obesity prevalence across the socioeconomic spectrum. These programs should incorporate actions that include consideration of the differential patterning of obesity by age, gender, and SES.

This study has several limitations. First, due to limitations in the available data, the model assumes that individuals do not transition between socioeconomic strata, and that mortality rates do not vary by BMI category. In order to overcome these limitations, future efforts should focus on improving the collection of information by gender, age, mortality, SES, and BMI. Second, given the data availability in the surveys, the TRs by BMI categories, age, and SES were calculated using only two data points and assumed to apply into the future through 2050. Future studies should incorporate more data points, collected over longer time horizons, to validate the model. Third, in the model, the TRs by age, gender, SES, and BMI category do not change over time. Future progressions to the model could include simulating obesity dynamics using TRs that change over time and that are affected by factors associated with each group of individuals according to their stratification by age, gender, SES, and BMI category. Fourth, in this study, educational attainment was used as a proxy of SES. Future studies should analyze the effect of other measures, such as HDI and wealth index, in the obesity transition to complement the results of this paper. Lastly, this study includes only urban areas (SALURBAL cities over 100 000 inhabitants) and patterns could be very different in rural areas.

## 5 Conclusion

This study illustrates how the obesity dynamics in Colombia and Mexico might change over time and how these countries could evolve through the different stages of the obesity transition. In particular, the findings show that Colombia is moving to stage 3 of the obesity transition, which is characterized by the greatest burden of obesity in the lowest SES population. On the other hand, Mexico was observed to be transitioning between stages 2 and 3, where the greatest burden of obesity is among low SES women and high SES men, while the greatest increases in obesity prevalence are projected to occur among high SES women and low SES men.

This study presents several novel contributions that set it apart from existing research. Firstly, this is the first study to apply system dynamics modeling to compare obesity transitions across multiple countries, with a particular focus on middle-income nations where data is often limited. Secondly, it is the first to compare the dynamics of obesity and to evaluate the obesity transition between Colombia and Mexico—two countries with significant differences in obesity prevalence. In Latin America and the Caribbean, Mexico has the highest age-standardized obesity prevalence among both men and women, while Colombia reports the lowest prevalence among men [70]. Lastly, the system dynamics model aligns with the conceptual framework of obesity transition by Jaacks et al. [14], enabling empirical and dynamic evaluation of whether a country is undergoing an obesity transition.

The model could be further extended and used to predict how other countries evolve through the different stages of the obesity transition with changes in economic development. Importantly, the findings highlight the differential patterning of obesity prevalence across population subgroups and the risk posed to these groups by the obesity transition. As such, the findings can be used to help prioritize public health interventions to prevent obesity with equity criteria in Colombia and Mexico.

## CRediT authorship contribution statement

**Jose D. Meisel:** Writing – review & editing, Writing – original draft, Validation, Software, Methodology, Formal analysis, Conceptualization. **Valentina Esguerra:** Writing – review & editing, Writing – original draft, Validation, Software, Formal analysis. **Carolina Pérez Ferrer:** Writing – review & editing, Validation, Formal analysis. **Ivana Stankov:** Writing – review & editing, Writing – original draft, Validation, Formal analysis. **Felipe Montes:** Writing – review & editing, Validation, Formal analysis. **Natalia Tumas:** Writing – review & editing, Validation, Formal analysis. **Usama Bilal:** Writing – review & editing, Validation, Formal analysis, Data curation. **Juan A. Valdivia:** Writing – review & editing, Writing – original draft, Validation, Software, Formal analysis. **Ana V. Diez Roux:** Writing – review & editing, Validation, Funding acquisition, Formal analysis, Data curation. **Olga L. Sarmiento:** Writing – review & editing, Writing – original draft, Validation, Supervision, Methodology, Funding acquisition, Formal analysis.

## **Funding**

The Salud Urbana en América Latina (SALURBAL)/Urban Health in Latin America Project is funded by the Wellcome Trust, UK [grant 205177/Z/16/Z]. For the purpose of open access, the author has applied a CC-BY public copyright license to any Author Accepted Manuscript version arising from this submission. JDM was funded by the Research office at Universidad de Ibague (Project number 24-007-ESP). JAV was funded by CEDENNA through the National Agency for Research and Development (ANID)/PIA/BASAL under grant number AFB220001. UB was also supported by the Office of the Director of the National Institutes of Health under award number DP5OD26429. The funding sources had no role in the analysis, writing or decision to submit the manuscript.

## Data availability statement

Data associated with the study has not been deposited into a publicly available repository. The datasets generated during and/or analysed during the current study are available from the corresponding author on reasonable request. Requests for the harmonized data set can be obtained by contacting the SALURBAL project salurbal.data@drexel.edu and after completing a data use agreements. Requests are reviewed by the Data Methods Core and Publications & Presentations Committee on a monthly basis. To learn more about SALURBAL’s data set, visit https://drexel.edu/lac/ or contact the project at salurbal@drexel.edu.

## Data acknowledgement statement

SALURBAL acknowledges the contributions of many different agencies in generating, processing, facilitating access to data or assisting with other aspects of the project. Please visit https://drexel.edu/lac/data-evidence for a complete list of data sources. The findings of this study and their interpretation are the responsibility of the authors and do not represent the views or interpretations of the institutions or groups that compiled, collected, or provided the data. The use of data from these institutions does not claim or imply that they have participated in, approved, endorsed, or otherwise supported the development of this publication. They are not liable for any errors, omissions or other defect or for any actions taken in reliance thereon.

## Ethics approval

The SALURBAL study protocol was approved by the Drexel University Institutional Review Board with ID #1612005035 and by appropriate site-specific IRBs.

## Declaration of competing interest

The authors declare that they have no known competing financial interests or personal relationships that could have appeared to influence the work reported in this paper.
