# Supplementary material for: Understanding the obesity dynamics by socioeconomic status in Colombian and Mexican cities using a system dynamics model
Source: Heliyon. 2024 Oct 29;10(22):e39921. doi: 10.1016/j.heliyon.2024.e39921 (PMC11600054; doi:10.1016/j.heliyon.2024.e39921)
Supplement: Multimedia component 1 [file mmc1.docx]

**Supplementary File**

**Supplement to: Understanding the obesity dynamics by socioeconomic status in Colombia and Mexico using a system dynamics model**

**Table of contents**

**Section 1. Estimation of transferences rates between Body mass index categories for each age group, gender, and SES group** 1

**Results of the heuristic** 2

**Section 2. Validation of the model** 3

**Section 3. Key assumptions of the system dynamics model** 8

**References** 8

**Section 1. Estimation of transferences rates between Body mass index categories for each age group, gender, and SES group**

We used a heuristic, which was based on previously published research (Meisel et al., 2016, 2018, 2020), to estimate the transference rates (TRs) between body mass index (BMI) categories by age group, gender, and SES using historical data obtained from the 2010 and 2015 ENSIN Survey, and the 2012 and 2016 ENSANUT Survey for Colombia and Mexico, respectively. We grouped the data into three BMI categories, within age groups of five years for Colombia and four years for Mexico. The heuristic is then used to approximates the prevalence rates by BMI category in 2015 for Colombia and 2016 for Mexico for each age group *i*, gender *j*, and SES group *k* independently using the following system of equations for each country:

$$P_{i+1, j,k}^{'}=B_{i,j,k}.P_{i,j,k} (1)$$

where

$B_{i,j,k}=A_{i,j,k}.(A_{i,j,k}.\left( A_{i,j,k}.\left( A_{i,j,k}.A_{i,j,k} \right) \right))$;

$A_{i,j,k}=\left( \begin{matrix} \alpha_{1,i,j,k}-\tau_{1,i,j,k} & \tau_{4,i,j,k} & 0 \\ \tau_{1,i,j,k} & \alpha_{2,i,j,k}-\tau_{2,i,j,k}-\tau_{4,i,j,k} & \tau_{3,i,j,k} \\ 0 & \tau_{2,i,j,k} & \alpha_{3,i,j,k}-\tau_{3,i,j,k} \end{matrix} \right)$; $P_{i+1,j,k}^{'}=\left( \begin{matrix} p_{N2, i+1,j,k}^{'} \\ p_{W2,i+1,j,k}^{'} \\ p_{O2,i+1,j,k}^{'} \end{matrix} \right)$;

$P_{i,j,k}=\left( \begin{matrix} P_{N1,i,,j,k} \\ P_{W1,i,j,k} \\ P_{O1,i,j,k} \end{matrix} \right)$;

where *i* ∈ (0,….,n) represents the age groups (for Colombia, the model used a total of 12 age groups, divided into intervals of 5 years, and for Mexico, the model used 15 age groups in intervals of 4 years); *j* ∈ (1, 2) represents gender (men and women, respectively); *k* ∈ (1, 2, 3) represents the SES groups (1= *less than primary completed or primary completed*, 2= *secondary completed*, and 3= *university completed*); *P_N1,i,,j,k_*, *P_W1,i,j,k_*, and *P_O1,i,,j,k_* are the prevalence rates by BMI category in 2010 for Colombia and 2012 for Mexico for age group *i,* gender *j,* and SES group *k*; *α_1,i,j,k_, α_2,i,j,k_,* and *α_3,i,j,k_* are the retention rates for individuals by BMI category, age group *i,* gender *j,* and SES group *k*, corresponding to the fraction of individuals who remain in the same BMI category between 2010 and 2015 for Colombia and 2012 and 2016 for Mexico; *τ_1,i,j,k_*, *τ_2,i,j,k_* *τ_3,i,j,k_*, and *τ_4,i,j,k_* are the TRs that are used to run the SD model; and *P’_N2,i+1,j,k_*, *P’_W2,i+1,j,k_*, and *P’_O2,i+1,j,k_* are the estimated prevalence rates by BMI category in 2015 for Colombia and 2016 for Mexico for each age group *i,* gender *j,* and SES group *k*. The matrix *A_i,j,k_* represents the equations used to calculate the estimated prevalence rates by BMI category in the year *t+1* for age group *i,* gender *j,* and SES group *k*.

Then, the heuristic calculates the TRs by BMI category for each age group, gender, and SES group by minimizing the quadratic difference between prevalence rates by BMI category, age, gender, and SES informed by health surveys (ENSIN and ENSANUT) in 2015 and 2016 for Colombia and Mexico, respectively, and the prevalence rates estimated by the SD model in the same years for each country. Specifically, the TRs were estimated by solving the system of equations (1) for each age group *i*, gender *j*, and SES group *k*, and minimizing the following equation:

$${Min QD}_{i, j,k}=\left[ \left( P_{N2,i+1,j,k}-p_{N2,i+1,j,k}^{'} \right)^{2}+\left( P_{W2,i+1,j,k}-p_{W2,i+1,j,k}^{'} \right)^{2}+\left( P_{O2,i+1,j,k}-p_{O2,i+1,j,k}^{'} \right)^{2} \right], (2)$$

with the restrictions

$$\alpha_{1,i,j,k}+\tau_{1,i,j,k}=1$$

$$\alpha_{2,i,j,k}+\tau_{2,i,j,k}+\tau_{4,i,j,k}=1$$

$\alpha_{3,i,j,k}+\tau_{3,i,j,k}=1$ (3)

$$0\leq\alpha_{l,i,j,k}\leq1, (l=1, 2, 3)$$

$$0\leq\tau_{m,i,j,k}\leq1, \left( m=1, 2, 3, 4 \right)$$

Where, *P_N2,i+1,i,j,k_*, *P_W2,i+1,i,j,k_*, and *P_O2,i+1,i,j,k_* are the prevalence rates by BMI category in 2015 for Colombia and 2016 for Mexico for age group *i+1,* gender *j,* and SES group *k*.

## **Results of the heuristic**

The results of the heuristic showed that the quadratic differences between the prevalence rates by BMI category reported by the health surveys and the estimated prevalence rates predicted by the heuristic, were equal to or less than 5% for each age group, gender, and SES group for both countries (Tables S1 and S2). Therefore, the results indicate that the prevalence rates predicted by the model closely replicate the prevalence rates observed in health surveys (ENSIN and ENSANUT).

**Table S1. Quadratic differences between the prevalence rates by BMI categories reported by the ENSIN in 2015 and the estimated 2015 prevalence rates using the heuristic, by age group, gender, and SES for Colombia**

|  | ***Male*** | | | ***Female*** | | |
| --- | --- | --- | --- | --- | --- | --- |
| **Age group** | ***Lower SES***  ***(%)*** | ***Middle SES***  ***(%)*** | ***Higher SES***  ***(%)*** | ***Lower SES***  ***(%)*** | ***Middle SES***  ***(%)*** | ***Higher SES***  ***(%)*** |
| 0-4 | 0.40 | 0.72 | 1.14 | 2.41 | 0.61 | 0.45 |
| 5-9 | 0.01 | 0.02 | 0.00 | 1.65 | 0.06 | 0.01 |
| 10-14 | 0.00 | 0.02 | 0.00 | 0.04 | 0.29 | 0.14 |
| 15-19 | 1.41 | 0.91 | 1.07 | 4.73 | 1.69 | 0.89 |
| 20-24 | 1.24 | 1.36 | 2.17 | 4.46 | 2.81 | 1.71 |
| 25-29 | 1.66 | 0.94 | 1.12 | 5.16 | 1.65 | 1.52 |
| 30-34 | 1.25 | 0.97 | 0.88 | 1.45 | 1.26 | 1.02 |
| 35-39 | 0.72 | 0.70 | 0.29 | 1.12 | 1.05 | 1.19 |
| 40-44 | 0.57 | 0.43 | 0.83 | 0.92 | 1.03 | 0.92 |
| 45-49 | 1.08 | 0.57 | 0.47 | 0.55 | 0.90 | 0.71 |
| 50-54 | 0.62 | 0.60 | 0.75 | 0.27 | 0.43 | 0.11 |
| 55-59 | 1.10 | 0.43 | 0.14 | 0.11 | 0.13 | 0.55 |

**Table S2. Quadratic differences between the prevalence rates by BMI categories reported by the ENSANUT in 2016 and the estimated 2016 prevalence rates using the heuristic, by age group, gender, and SES for Mexico**

|  | ***Male*** | | | ***Female*** | | |
| --- | --- | --- | --- | --- | --- | --- |
| **Age group** | ***Lower SES***  ***(%)*** | ***Middle SES***  ***(%)*** | ***Higher SES***  ***(%)*** | ***Lower SES***  ***(%)*** | ***Middle SES***  ***(%)*** | ***Higher SES***  ***(%)*** |
| 0-3 | 0.60 | 0.76 | 0.42 | 0.24 | 0.23 | 0.45 |
| 4-7 | 1.09 | 0.12 | 0.56 | 1.45 | 0.21 | 0.13 |
| 8-11 | 0.12 | 0.02 | 0.33 | 0.03 | 0.10 | 0.18 |
| 12-15 | 0.15 | 0.04 | 0.04 | 0.56 | 0.00 | 1.02 |
| 16-19 | 0.23 | 1.22 | 0.03 | 1.45 | 0.14 | 0.33 |
| 20-23 | 1.35 | 0.52 | 0.37 | 1.93 | 0.18 | 1.24 |
| 24-27 | 0.56 | 0.18 | 0.78 | 0.00 | 0.09 | 1.35 |
| 28-31 | 1.11 | 0.19 | 1.20 | 0.24 | 0.43 | 1.05 |
| 32-35 | 0.22 | 0.37 | 1.21 | 0.51 | 0.22 | 0.10 |
| 36-39 | 0.19 | 0.00 | 0.74 | 0.92 | 0.04 | 0.07 |
| 40-43 | 0.20 | 0.00 | 0.06 | 0.31 | 0.08 | 0.20 |
| 44-49 | 0.08 | 0.00 | 1.45 | 0.21 | 0.26 | 0.07 |
| 50-53 | 0.24 | 0.09 | 1.31 | 0.05 | 0.00 | 0.27 |
| 54-57 | 0.18 | 0.01 | 0.16 | 0.08 | 0.31 | 0.13 |
| 58-61 | 0.65 | 0.48 | 0.80 | 0.00 | 0.09 | 0.22 |

**Section 2. Key assumptions of the system dynamics model**

In the table S3, we summarize the key assumptions used in the system dynamics model.

**Table S3. Key assumptions used in the system dynamics model**

| **Key assumptions** | **Description** |
| --- | --- |
| No transition between socioeconomic strata | There are no available data regarding transition rates between socioeconomic strata by BMI category, age, gender, and SES |
| The mortality rate for each BMI category in each age group is the same | There are no available data regarding mortality rates by BMI category, age, gender, and SES |
| TRs (derived by comparing the two time points in each country) are stable over the simulated timeframe. TRs by age, gender, SES, and BMI category do not change over time. | We lack of additional longitudinal data needed to calculate the TRs by BMI category, age, gender, and SES from one year to the next. |

**Section 3. Validation of the model**

We used a behaviour reproduction method to test whether the SD model can reproduce the behaviour of the system. We used a two-sample Kolmogorov–Smirnov test (Corder & Foreman, 2014) to validate whether the model has been able to reproduce the prevalence rates by age group for each BMI category, gender, and SES group observed in the data. The two-sample Kolmogorov–Smirnov test checks whether the two data samples (the simulated data and the survey data) come from populations with the same distribution. The null hypothesis is that the two data samples come from a population with the same distribution. Due to a lack of additional longitudinal data needed to calculate the TRs by BMI category, age, gender, and SES from one year to the next, we validate the behaviour reproduction of the model for the year 2015 for Colombia and 2016 for Mexico.

We used the two-sample Kolmogorov–Smirnov test function in Mathematica to compare the prevalence rates by BMI category, age, gender, and SES reported by the health surveys in 2015 for Colombia (ENSIN) and 2016 for Mexico (ENSANUT) and the prevalence rates estimated by the SD model in the same years for each country. The results of the tests show that the SD model is able to reproduce the prevalence rates by age for each BMI category, gender, and SES (all p-values were greater than 0.05, therefore, the null hypothesis (Ho) cannot be rejected, suggesting that the two data samples, come from populations with the same distribution) (Tables S4 and S5, and Fig. S1 and S2).

**Table S4. Results of the Kolmogorov–Smirnov tests for Colombia by gender and SES**

|  | **Men** | | **Women** | |
| --- | --- | --- | --- | --- |
| **Level** | **Item** | **p-value** | **Item** | **p-value** |
| Lower SES | Not-overweight prevalence rates vs. estimated prevalence rates of not-overweight by age | 0.1 | Not-overweight prevalence rates vs. estimated prevalence rates of not-overweight by age | 0.1 |
|  | Overweight prevalence rates vs. estimated prevalence rates of overweight by age | 0.869 | Overweight prevalence rates vs. estimated prevalence rates of overweight by age | 0.869 |
|  | Obesity prevalence rates vs. estimated prevalence rates of obesity by age | 0.1 | Obesity prevalence rates vs. estimated prevalence rates of obesity by age | 0.1 |
| Middle SES | Not-overweight prevalence rates vs. estimated prevalence rates of not-overweight by age | 0.256 | Not-overweight prevalence rates vs. estimated prevalence rates of not-overweight by age | 0.869 |
|  | Overweight prevalence rates vs. estimated prevalence rates of overweight by age | 0.536 | Overweight prevalence rates vs. estimated prevalence rates of overweight by age | 0.536 |
|  | Obesity prevalence rates vs. estimated prevalence rates of obesity by age | 0.256 | Obesity prevalence rates vs. estimated prevalence rates of obesity by age | 0.536 |
| Higher SES | Not-overweight prevalence rates vs. estimated prevalence rates of not-overweight by age | 0.256 | Not-overweight prevalence rates vs. estimated prevalence rates of not-overweight by age | 0.536 |
|  | Overweight prevalence rates vs. estimated prevalence rates of overweight by age | 0.998 | Overweight prevalence rates vs. estimated prevalence rates of overweight by age | 0.998 |
|  | Obesity prevalence rates vs. estimated prevalence rates of obesity by age | 0.256 | Obesity prevalence rates vs. estimated prevalence rates of obesity by age | 0.256 |

**Table S5. Results of the Kolmogorov–Smirnov tests for Mexico by gender and SES**

|  | **Man** | | **Women** | |
| --- | --- | --- | --- | --- |
| **Level** | **Item** | **p-value** | **Item** | **p-value** |
| Lower SES | Not-overweight prevalence rates vs. estimated prevalence rates of not-overweight by age | 0.999 | Not-overweight prevalence rates vs. estimated prevalence rates of not-overweight by age | 0.678 |
|  | Overweight prevalence rates vs. estimated prevalence rates of overweight by age | 0.386 | Overweight prevalence rates vs. estimated prevalence rates of overweight by age | 0.678 |
|  | Obesity prevalence rates vs. estimated prevalence rates of obesity by age | 0.932 | Obesity prevalence rates vs. estimated prevalence rates of obesity by age | 0.938 |
| Middle SES | Not-overweight prevalence rates vs. estimated prevalence rates of not-overweight by age | 0.938 | Not-overweight prevalence rates vs. estimated prevalence rates of not-overweight by age | 0.999 |
|  | Overweight prevalence rates vs. estimated prevalence rates of overweight by age | 0.938 | Overweight prevalence rates vs. estimated prevalence rates of overweight by age | 0.678 |
|  | Obesity prevalence rates vs. estimated prevalence rates of obesity by age | 0.678 | Obesity prevalence rates vs. estimated prevalence rates of obesity by age | 0.938 |
| Higher SES | Not-overweight prevalence rates vs. estimated prevalence rates of not-overweight by age | 0.938 | Not-overweight prevalence rates vs. estimated prevalence rates of not-overweight by age | 0.999 |
|  | Overweight prevalence rates vs. estimated prevalence rates of overweight by age | 0.999 | Overweight prevalence rates vs. estimated prevalence rates of overweight by age | 0.938 |
|  | Obesity prevalence rates vs. estimated prevalence rates of obesity by age | 0.386 | Obesity prevalence rates vs. estimated prevalence rates of obesity by age | 0.938 |


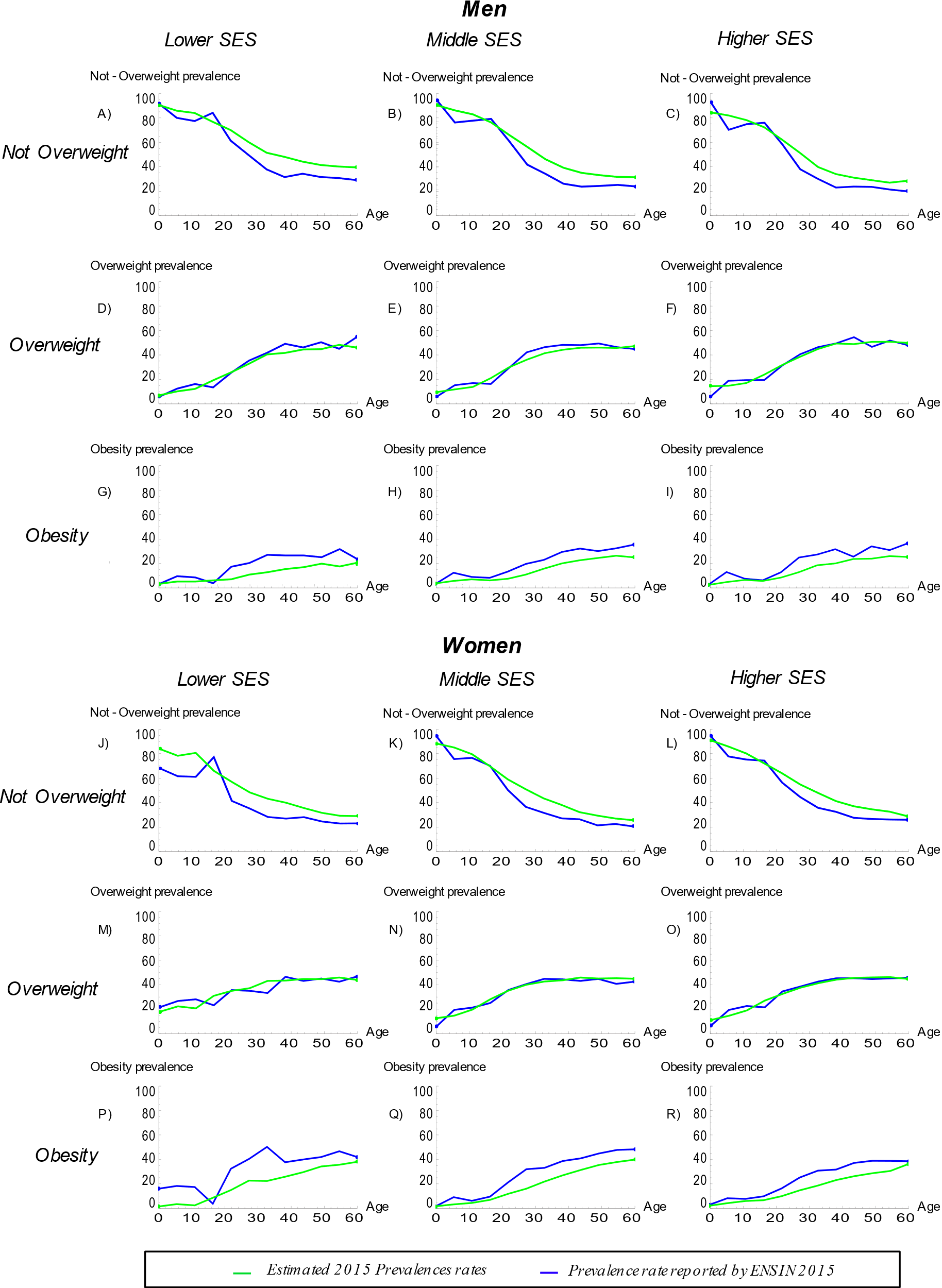


**Fig S1. Prevalence rates by age reported by ENSIN in 2015 vs. estimated 2015 prevalence rates by age using the SD model by BMI categories, gender, and SES for Colombia.** (A, E, I) Lower SES; (B, F, J) Middle SES; (C, G, K); and Higher SES. Blue= Prevalence rates by age reported by ENSIN in 2015; Green= Estimated 2015 prevalence rates by age using the SD model.


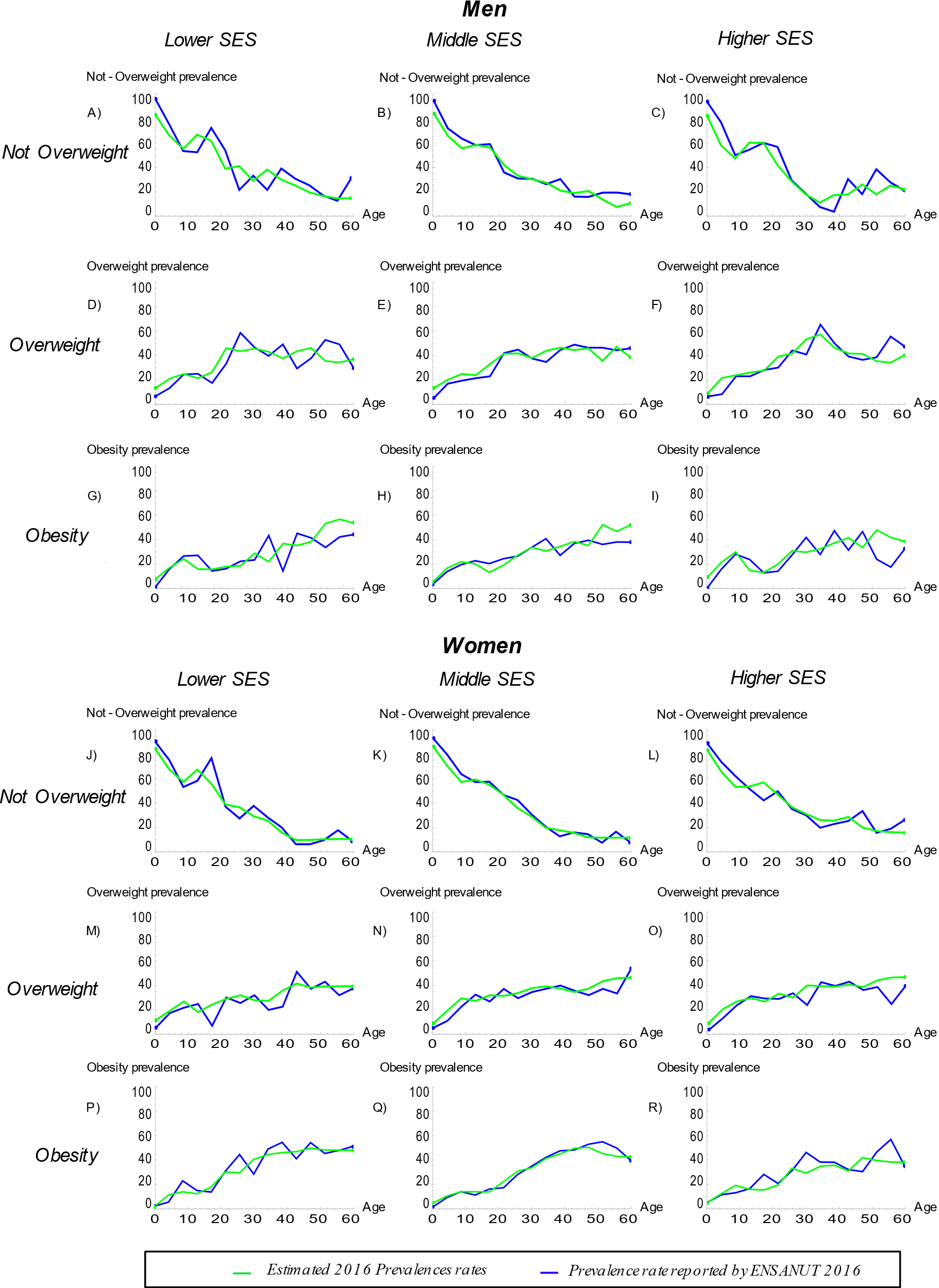


**Fig S2. Prevalence rates by age reported by ENSANUT in 2016 vs. estimated 2016 prevalence rates by age using the SD model by BMI categories, gender, and SES for Mexico.** (A, E, I) Lower SES; (B, F, J) Middle SES; and (C, G, K) Higher SES. Blue= Prevalence rates by age reported by ENSANUT in 2016; Green= Estimated 2016 prevalence rates by age using the SD model.

**References**

Corder, G. W., & Foreman, D. I. (2014). *Nonparametric Statistics: A Step-by-Step Approach* (2 edition). Wiley.

Meisel, J. D., Ramirez, A. M., Esguerra, V., Montes, F., Stankov, I., Sarmiento, O. L., & Valdivia, J. A. (2020). Using a system dynamics model to study the obesity transition by socioeconomic status in Colombia at the country, regional and department levels. *BMJ Open*, *10*(6), e036534. https://doi.org/10.1136/bmjopen-2019-036534

Meisel, J. D., Sarmiento, O. L., Olaya, C., Lemoine, P. D., Valdivia, J. A., & Zarama, R. (2018). Towards a novel model for studying the nutritional stage dynamics of the Colombian population by age and socioeconomic status. *PLOS ONE*, *13*(2), e0191929. https://doi.org/10.1371/journal.pone.0191929

Meisel, J. D., Sarmiento, O. L., Olaya, C., Valdivia, J. A., & Zarama, R. (2016). A system dynamics model of the nutritional stages of the Colombian population. *Kybernetics*, *45*(4), 554–570. http://dx.doi.org/10.1108/K-01-2015-0010
